# Emerging pathways in thromboinflammation of sickle cell disease: novel findings in disease pathogenesis

**DOI:** 10.1016/j.rpth.2025.103193

**Published:** 2025-09-24

**Authors:** Nirupama Ramadas, Erica Sparkenbaugh

**Affiliations:** 1 Blood Research Center, School of Medicine, University of North Carolina at Chapel Hill, Chapel Hill, North Carolina, USA; 2 Department of Pathology and Laboratory Medicine, School of Medicine, University of North Carolina at Chapel Hill, Chapel Hill, North Carolina, USA

**Keywords:** sickle cell disease, hemoglobinopathy, thromboinflammation, anemia, platelets, neutrophils

## Abstract

Sickle cell disease (SCD) is increasingly recognized as a chronic thromboinflammatory disorder, marked by persistent intravascular hemolysis, sustained endothelial activation, and multicellular aggregate formation. Free hemoglobin and heme act as damage-associated molecular patterns, activating Toll-like receptor 4 and promoting inflammatory and procoagulant responses in endothelial cells, monocytes, and neutrophils. Hyperreactive platelets, sickled red blood cells, and activated leukocytes interact with the endothelium to propagate vascular occlusion and thrombin generation. This persistent thromboinflammatory state targets low-flow microvascular beds in the bone marrow, lung, kidney, spleen, and brain, culminating in progressive end-organ dysfunction. In this article, we review the cellular and molecular drivers of thromboinflammation in SCD, highlighting how sustained vascular injury leads to deep vein thrombosis, stroke, nephropathy, and cardiopulmonary complications. Understanding these mechanisms is essential for developing targeted strategies to disrupt the thromboinflammatory cycle and prevent irreversible organ damage in SCD.

## Essentials

• A β-globin mutation causes sickle cell disease, leading to hemolysis and vaso-occlusion.
• Free heme activates leukocytes, platelets, and endothelial cells, causing inflammation.
• RBCs, neutrophils, and platelets aggregate, causing occlusion, coagulation, and inflammation.
• Thromboinflammation drives thrombosis, stroke, kidney injury, and cardiopulmonary disease.

## 1 Introduction

### 1.1 Genetic basis and history of sickle cell disease

Sickle cell disease (SCD) comprises a group of inherited hemoglobinopathies caused by a single point mutation in the β-globin gene. Adult hemoglobin (HbA) is a tetramer of 2 α-globin and 2 β-globin subunits. In SCD, a single-nucleotide substitution of adenine (A) to thymine (T) in the sixth codon results in an amino acid substitution of a glutamic acid for a valine, generating the sickle hemoglobin (HbS) allele. HbS with 2 β^S^ subunits (HbSS) will form abnormal polymers under deoxygenated conditions, causing red blood cells (RBCs) to undergo shape change to a crescent or sickle shape. These deformed cells undergo hemolysis, leading to anemia. Moreover, they can block small blood vessels, leading to vaso-occlusive events (VOE) that result in pain crises and end-organ damage [1]. The most common and phenotypically severe forms of the disease result from homozygous inheritance of the HbSS allele or compound heterozygosity of HbS with a β-thalassemia null mutation (HbSβ^0^). Other variants, such as HbSC and HbS-β+ thalassemia (HbSβ^+^) are generally less phenotypically severe [1].

The first documented case of SCD was in the early 1900s, when Walter Clement Noel, a dental student from Grenada living in Chicago, presented with pneumonia-like symptoms that we would now recognize as acute chest syndrome (ACS). His case was described by 2 physicians, Drs Ernest Irons and James Herrick, who observed anemia and irregular, elongated RBCs in the blood smear [2]. Similar cases were reported over the next few years. By 1927, Hanh and Gillespie reported that RBC sickling was due to hypoxia [3]. In 1949, James Neel [4] noted the inheritability of the disease, and in the same year, Linus Pauling described it as a molecular disease, when he discovered that the electrophoretic mobility of HbS was distinct from HbA [5]. The amino acid substitution was described in 1957 by Vernon Ingram and John Hunt, and by 1959, the mutation was mapped to the sixth codon [3]. Despite these early advances in describing the molecular basis of SCD, therapeutic options have been slow to arrive.

The current standard treatments for SCD include chronic blood transfusions and hydroxyurea (HU). HU increases fetal hemoglobin levels by inducing γ-globin expression, thereby reducing red cell sickling. Between 2017 and 2019, 3 additional drugs were approved: L-glutamine (an antioxidant), crizanlizumab (an anti-P-selectin antibody), and voxelotor (an HbS polymerization inhibitor). Voxelotor was voluntarily withdrawn from the market in 2024 due to an unfavorable risk-benefit profile [6]. The European Medicines Agency revoked approval of crizanlizumab in 2023 after it failed to demonstrate efficacy in the Study of Two Doses of Crizanlizumab Versus Placebo in Adolescent and Adult Sickle Cell Disease Patients (STAND) Phase III trial, although it remains in use in the United States [7]. Most recently, 2 cell-based gene therapies were approved in December 2023. Casgevy uses Clustered Regularly Interspaced Short Palindromic Repeats (CRISPR) Cas9 to disable B cell lymphoma transcriptio factor a (*BCL11a*), a repressor of γ-globin expression, leading to fetal hemoglobin expression [8]. Lyfgenia uses a lentiviral vector to induce expression of an antisickling hemoglobin A variant [9]. Both therapies have demonstrated an 88% to 94% reduction in the incidence of painful crises in clinical trials [10].

### 1.2 Incidence, global burden, and clinical manifestations

The sickle cell mutation arose spontaneously in sub-Saharan Africa and South Asia, because heterozygous HbAS is an evolutionary response to malaria [11]. Today, SCD affects an estimated 7 to 10 million individuals, with about 500,000 babies born annually with the disease, an overwhelming majority in sub-Saharan Africa [12]. SCD remains a leading cause of all-cause mortality, especially in children under the age of five [12]. In the United States, the incidence is 1 in 365 African Americans with approximately 100,000 people living with the disease [12]. Even with improved pediatric care—including early introduction of antibiotics and hydroxyurea—the median age of death is just 43 years old [13] and lifetime medical costs exceed $1.5 million per patient [14].

In this review, we will explore the well-characterized pathophysiology of SCD through the lens of thromboinflammation. While recent reviews have thoroughly covered this topic [1,15,16], our goal is to highlight recent novel findings in this field and discuss how thromboinflammatory mechanisms contribute to the progression of organ failure. Nearly every organ system is affected by both acute and chronic complications, and the clinical manifestations of the disease have been well-documented by others [1]. A summary is provided in Table [1,17] .

**Table:** Summary of acute and chronic complications in sickle cell disease [1,17].

System	Acute complications	Chronic complications
Cerebrovascular	Ischemic stroke	Neurocognitive decline, intracranial hemorrhage, and ischemic brain injury
Musculoskeletal	Acute vaso-occlusive episodes, dactylitis, and aseptic necrosis	Chronic pain, tender back, and decreased range of motion
Circulatory	Splenic sequestration	Functional asplenia and repeated splenic infarction
Cardiopulmonary	Acute myocardial infarction and acute chest syndrome	Chronic chamber enlargement and pulmonary hypertension
Ocular	Vitreous hemorrhage	Proliferative retinopathy, decreased vision, and blindness
Renal	Hematuria, acute glomerular nephritis, andpriapism	Chronic kidney injury and renal failure
Gastrointestinal and hepatic	Hepatic ischemia, benign cholestasis, transfusional iron overload, and abdominal pain	Hepatic sequestration crisis, chronic cholelithiasis, hepatopathy, and gall stones
Infection	Bacteremia-induced leukocytosis, aplastic or hypoproliferative crisis, and meningitis	Sepsis, bacterial pneumonia, and viral-induced pulmonary infection
Bone	Bone pain	Bone infarcts, avascular necrosis of femoral humeral head, and osteomyelitis
Hematological	Myofascial syndrome, hemolytic anemia, reticulocytosis, and low Hb	Leg ulceration
Psychosocial	Anxiety and pain	Depression, low self-esteem, social isolation
Growth and development	Nutritional deficiency and increased energy expenditure	Impaired growth and delayed puberty and menarche

## 2 Thromboinflammation and Immunothrombosis

The innate immune and hemostatic systems are essential for responding to both external pathogens and internal injury. Although traditionally studied as separate processes, research over the past 2 decades has revealed significant crosstalk between these 2 systems. Two terms—immunothrombosis and thromboinflammation—have been introduced to describe these interactions.

Immunothrombosis was a term first introduced by Engelmann and Massberg [18] to refer to a host defense mechanism in which innate immune cells activate coagulation pathways in response to pathogen-associated molecular patterns or damage-associated molecular patterns (DAMPs) [18]. For example, monocytes increase the expression of tissue factor (TF) to initiate extrinsic coagulation activation, while neutrophils release neutrophil extracellular traps (NETs), which contribute to both extrinsic and intrinsic coagulation activation.

Thromboinflammation describes a closely related phenomenon, often conceptualized as thrombosis accompanied by an inflammatory response. It may result from the loss of the normal antithrombotic and antiinflammatory responses of endothelial cells, combined with activation of proinflammatory signaling pathways by coagulation proteases through protease-activated receptors (PARs). This creates a pathologic feedback loop that exacerbates both thrombotic and inflammatory responses [19].

## 3 Overview of the Pathophysiology OF SCD

Although caused by a single-nucleotide mutation, the downstream consequences of SCD are multifactorial and complex (Figure). In this section, we describe how coagulation activation, RBCs, endothelial cells, leukocytes, and platelets interact to drive inflammation and vaso-occlusion.

**Figure:**
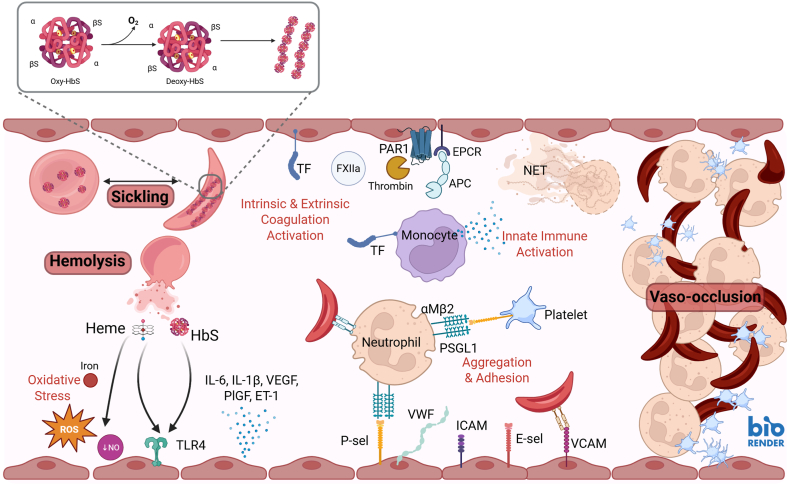
Cellular and molecular actions involved in sickle cell disease (SCD) thromboinflammation. Sickle hemoglobin (HbS) undergoes abnormal polymerization after deoxygenation, leading to sickling and hemolysis of red blood cells. Release of free hemoglobin and heme into circulation depletes nitric oxide. They also act as DAMPs that activate TLR4 on endothelial cells, monocytes, neutrophils, and platelets. This increases release of cytokines (interleukin [IL]-6 and IL-1β), growth factors (vascular endothelial growth factor [VEGF] and placental growth factor [PlGF]), and endothelin (ET)-1. ET-1 activation upregulates adhesion molecules E-selectin (E-sel), P-selectin (P-sel), von Willebrand factor (VWF), intracellular adhesion molecule (ICAM), and vascular cell adhesion molecule (VCAM). Neutrophils upregulate P-selectin glycoprotein ligand (PSGL)1, which binds P-selectin and mediates multicellular aggregate formation and vaso-occlusion. Concurrently, upregulation of tissue factor (TF) and activation of Factor (F)XII drive thrombin generation, which exacerbates platelet activation and contributes to endothelial activation and inflammation through protease-activated receptor (PAR)1 signaling. Neutrophils also support thrombin generation and platelet adhesion through release of neutrophil extracellular traps (NETs). These chronic recurrent events ultimately drive end-organ damage and are significant contributes to morbidity and mortality in SCD.

### 3.1 Coagulation activation in SCD

The mechanisms of coagulation activation in SCD have been reviewed in depth elsewhere, but we will briefly summarize them in this section [15]. In the extrinsic pathway, formation of the TF–factor (F)VIIa complex initiates coagulation by activating FXa. The intrinsic pathway is triggered when FXII, bound to its cofactor high-molecular-weight kininogen (HK), is activated by negatively charged surfaces. FXIIa generates both FXIa and kallikrein, both of which activate FIXa. Both FVIIa and FIXa converge to activate FXa, which in complex with FVa, converts prothrombin to thrombin. Thrombin then cleaves fibrinogen to form fibrin, resulting in a clot. Notably, both extrinsic and intrinsic coagulation cascades are pathologically activated in SCD, contributing to a hypercoagulable state that intersects directly with inflammation and endothelial dysfunction [20].

SCD is one of the few pathologic conditions in which TF expression is abnormally induced on monocytes [21,22] and endothelial cells [[23], [24], [25]]. Preclinical models suggest that TF expressed on endothelial cells primarily drives inflammation, whereas monocyte-derived and extravascular TF plays a more significant role in thrombin generation [26,27]. Importantly, inhibition of TF, FXa, and thrombin has been shown to reduce thromboinflammation [28,29], organ damage [28], and microvascular stasis [30] in SCD. Notably, HU has been shown to normalize plasma markers of coagulation activation, TF expression, and endothelial activation in both adult and pediatric patients [31,32]. It also reduces both platelet and neutrophil counts [33], as well as markers of inflammation, neutrophil activation, and oxidative stress in patients with HbSS. These data suggest that hemolysis, coagulation, and inflammation are interconnected in this disease setting.

The intrinsic pathway is also increasingly recognized as a key player in SCD. In 2020, we reported that patients with SCD and Townes sickle mice have elevated levels of cleaved HK, indicating ongoing intrinsic activation in SCD. HK deficiency attenuated thromboinflammation and protected sickle mice from end-organ damage and mortality [34]. More recently, we found that patients with SCD have elevated circulating levels of FXIIa, FXIa, kallikrein, and FIXa bound to their plasma inhibitors at steady state [35]. Genetic deletion or pharmacologic inhibition of FXIIa attenuated thromboinflammation, arterial and venous thrombosis, and microvascular stasis in a mouse model of SCD [35]. Overall, our data suggest that both TF-dependent extrinsic coagulation and FXIIa-dependent intrinsic coagulation drive thromboinflammation and downstream complications in SCD.

Another contributor to the hypercoagulable state in SCD is the loss of natural anticoagulants. Plasma levels of protein C and its cofactor protein S are substantially reduced in patients with SCD, and they are further depleted after VOE or stroke [[36], [37], [38]]. This depletion may result from decreased hepatic production or increased consumption in the setting of ongoing coagulation activation. In normal conditions, protein C binds to the endothelial protein C receptor (EPCR), where it is activated by thrombin bound to thrombomodulin. Activated protein C (APC) dissociates from thrombomodulin and inactivates FVa and FVIIIa, with its anticoagulant activity enhanced by protein S. Recent studies have demonstrated reduced EPCR expression in the cerebra [39] and renal vasculature [40], suggesting that protein C activation may be impaired due to reduced bioavailability of EPCR. Loss of this regulatory pathway in SCD removes an important check on coagulation and promotes excess thrombin generation.

### 3.2 Complement cascade

Complement activation is a prominent feature of SCD, both at steady state and during vaso-occlusive crises [41,42]. Heme released during hemolysis triggers the alternative pathway [43], while ischemia-reperfusion injury and endothelial damage activate the lectin pathway [44]. In sickle mice, C5a infusion induces vascular stasis and inflammation [45], while blockade of the alternative (anti-MASP3) or lectin (anti-MASP2) pathways reduces heme-induced and ischemia-reperfusion–induced stasis [45]. These findings suggest that complement activation contributes directly to both inflammation and vascular occlusion. Ongoing clinical studies, including the Crovalimab for the Treatment and Prevention of Vaso-Occlusive Episodes in Sickle Cell Disease (CROSSWALK) trial of the C5 inhibitor crovalimab (NCT05075824), aim to translate these insights into therapies for patients with SCD.

### 3.3 Red blood cells

Deoxygenation of HbS leads to polymerization, causing RBC sickling and extravascular hemolysis. This releases cell-free hemoglobin and heme into circulation, where they act as DAMPs that activate Toll-like receptor (TLR)4 on both leukocytes [46] and endothelial cells [[47], [48], [49]]. In parallel, cell-free HbS and free iron scavenge nitric oxide (NO), reducing its bioavailability and promoting oxidative stress and inflammation [50,51]. Heme also acts independently of TLR4, via reactive oxygen species generation, leading to activation of innate immune cells [[52], [53], [54]].

Sickled RBCs (sRBCs) also undergo structural and biochemical changes that promote thrombosis and adhesion. The cells externalize phosphatidylserine (PS), a process linked to dehydration [55]. Recent evidence implicates the mechanosensor Piezo1 in this process: its activation leads to calcium influx through the Gardos channel, promoting PS exposure [56]. PS provides a procoagulant scaffold that not only promotes thrombin generation [36] but also enhanced cell–cell interactions. sRBCs express adhesion molecules such as intracellular adhesion molecule 4 and basal cell adhesion molecule, further promoting adhesion to the vasculature and other cells [57,58]. In addition to providing a procoagulant surface, sRBCs can release microvesicles that activate the intrinsic coagulation cascade, which contributes to thrombin generation in SCD [35,59].

### 3.4 Endothelial cells

Endothelial cells are central to the thromboinflammatory cascade in SCD. They become activated in response to several upstream signals. DAMPs such as HbS and heme engage TLR4 and promote proinflammatory signaling, leading to expression of several inflammatory cytokines such as tumor necrosis factor α, interleukin (IL)-1β, IL-6, IL-18, and chemokines such as monocyte chemoattractant protein 1 and IL-8 [47,60]. Angiogenic factors such as vascular endothelial growth factor (VEGF) and placental growth factor (PlGF) are also upregulated, contributing to vascular remodeling and inflammation [61,62]. Endothelial cells also produce endothelin (ET)-1, a potent vasoconstrictor that drives changes in vascular tone, ischemia, pulmonary hypertension (PH), and chronic kidney disease in SCD [63,64].

Beyond their inflammatory role, endothelial cells are critical sensors for the coagulation-independent functions of coagulation proteases, primarily via expression of PARs. The PAR family (PAR1-4) are G-protein–coupled receptors that are activated by proteolytic cleavage of an extracellular terminus, generating a tethered ligand that initiates signaling [65]. In this section, we focus on PAR1 and PAR2, the 2 most-studied PARs in SCD. PAR2 is activated by plasmin, tryptase, trypsin, FXa, and the TF-FVIIa complex. We previously reported that PAR2-deficient sickle bone marrow chimeras have reduced vascular inflammation, which mimicked what we observed with TF and FXa inhibition [29]. These findings suggests that endothelial TF-FVIIa and FXa contribute to vascular inflammation primarily through PAR2 activation.

In contrast, PAR1 is activated by matrix metalloproteases, plasmin, thrombin, and APC. In our studies, PAR1-deficient sickle bone marrow chimeras had no reduction in inflammation, consistent with the lack of effect observed when thrombin was inhibited with dabigatran [29]. This suggested that thrombin–PAR1 signaling does not drive inflammatory cytokine production in SCD. However, more recent studies found that thrombin–PAR1 signaling directly contributes to vascular stasis in SCD [30], suggesting that this pathway mediates endothelial activation and adhesion molecule expression, independent of inflammatory cytokine expression.

PAR1 is notable because of its biased agonism; its downstream signaling differs depending on which protease activates it and where it is cleaved. Indeed, activation of PAR1 by APC has beneficial, antiinflammatory effects [66]. We hypothesized that loss of PAR1 expression would eliminate the beneficial APC signaling. Supporting this, we found that inhibition of APC worsens thromboinflammatory outcomes in SCD mice [67]. Moreover, canonical thrombin–PAR1 signaling exacerbates inflammation, whereas noncanonical APC-PAR1 signaling suppresses it in sickle mice [67]. Complicating this dynamic is the reduced expression of EPCR in SCD, particularly in the renal [40] and cerebral vasculature [39]. Since EPCR is required for both APC generation and APC-PAR1 protective signaling, its loss not only impairs the anticoagulant protein C pathway but also removes a critical antiinflammatory brake. This highlights the context-dependent, finely balanced nature of PAR1 signaling in SCD, where both the availability of specific proteases and the integrity of EPCR expression dictate whether PAR1 signaling is protective or pathogenic [68].

Endothelial cells play a central role in vaso-occlusion in SCD through dysregulated release and processing of von Willebrand factor (VWF). Upon activation via TLR4 and PAR1 signaling, endothelial Weibel–Palade bodies release ultra-large VWF (ULVWF) multimers, which under shear stress, form adhesive strings that promote sickle RBC and leukocyte adhesion [69]. In SCD, impaired cleavage of ULVWF by **a** **d**isintegrin **a**nd **m**etalloprotease with a **t**hrombo**s**pondin type 1 motif, member **13 (**ADAMTS-13) exacerbates this process [70]. Mechanistically, this impairment is linked to elevated plasma levels of free hemoglobin and thrombospondin-1, both of which bind VWF and inhibit its cleavage. Notably, pharmacologic or genetic reduction of endothelial VWF, or administration of recombinant ADAMTS-13, attenuates vaso-occlusion, inflammation, hemolysis, and organ damage in multiple SCD mouse models, even when administered after an acute insult [[71], [72], [73], [74]]. A new study found that a subset of hepatic macrophages, Clec4f^+^-Marco^high^ macrophages, are critical for clearing VWF cleaved by ADAMTS-13 from the circulation of sickle mice [75]. This highlights ULVWF as both a mediator and a potential therapeutic target in VOE. Intriguingly, new data suggest that alternative proteases beyond ADAMTS-13 may also contribute to VWF cleavage in SCD, adding an additional layer of complexity to this thromboinflammatory axis [74].

### 3.5 Leukocytes

Leukocytes, particularly monocytes and neutrophils, are central amplifiers of thromboinflammation in SCD through direct cellular interactions, expression of procoagulant molecules, and the release of inflammatory mediators. Although leukocytes have many roles in the pathology of SCD, in this section, we focus on their direct contribution to thromboinflammation.

#### 3.5.1 Monocytes and macrophages

Similar to endothelial cells, HbS and free heme activate TLR4 on monocytes to increase cytokines (tumor necrosis factor α, IL-1β, and IL-6) and TF expression [25,49]. Indeed, TF-positive monocytes are correlated with inflammatory biomarkers in SCD [21,24], amplifying both endothelial and coagulation activation.

Interestingly, in the same study that highlighted the role of Clec4f^+^-Marco^high^ macrophages in clearing cleaved VWF from the circulation, this macrophage subset was also shown to remove sRBCs from the hepatic circulation [75]. This positions macrophages as both drivers and regulators of disease, with context-dependent roles in modulating thromboinflammation and vascular injury.

#### 3.5.2 Neutrophils

Neutrophils are key effectors of vaso-occlusion in SCD. Activated by heme, likely via TLR4 [60,76], the cells release NETs [77], chromatin structures containing histones, negatively charged DNA, and elastase. NETs not only contribute to vaso-occlusion but also serve as procoagulant scaffolds that provide a negatively charged surface that supports FXII autoactivation and downstream thrombin generation [78].

Activated neutrophils upregulate P-selectin-glycoprotein-ligand 1, which binds P-selectin on both endothelial cells and platelets to form the multicellular aggregates that cause vaso-occlusion [79]. SCD neutrophils also express higher levels of CD11b/CD18 (Mac1) than controls, promoting their adhesive nature [80]. Indeed, FXII binds to uPAR on neutrophils and upregulates CD11b/CD18 expression in SCD, linking coagulation with neutrophil-driven inflammation [35,81].

### 3.6 Platelets

In SCD, platelets are hyperactivated at steady state and during VOE [82,83]. This heightened activation has been linked to intravascular hemolysis, NO depletion [84], and activation of TLR4 by both high-mobility group box (HMGB)1 [85] and heme [86]. Additionally, thrombin activates platelets via PAR1 and PAR4 [65]. These stimuli trigger platelet degranulation and surface expression of adhesive and inflammatory molecules P-selectin, VWF, and CD40 ligand (CD40L), which mediate platelet binding to endothelium and leukocytes, facilitating the formation of multicellular aggregates that obstruct microvessels and promote vaso-occlusion. Moreover, platelets binding to neutrophils promotes NET release [87]. Activated platelets can also induce TF expression on monocytes, both through direct P-selectin binding [88] and via soluble CD40L signaling [83].

Another key pathway in platelet activation in SCD is ADP-P2Y_12_ receptor signaling. Platelet activation in response to hemolysis-derived heme [89] and thrombin triggers the release of adenosine diphosphate (ADP) from platelet dense granules. ADP acts in an autocrine and paracrine fashion to activate P2Y_12_, a G-protein–coupled receptors that mediates platelet aggregation, via stabilization of integrin α_IIb_β_3_ in its active conformation and promotes PS exposure [90]. These PS-expressing platelets represent a procoagulant subset that support thrombin generation and fibrin formation [91].

Despite the central role of platelets in both VOE and coagulation, clinical trials targeting platelets have been unsuccessful. Aspirin showed no effect on VOE incidence in pediatric and adult SCD cohorts [92,93]. Interestingly, the P2Y_12_ antagonist prasugrel reduced platelet activation biomarkers in both a preclinical mouse model [94] and patients [95] and reduced platelet-neutrophil aggregates *ex vivo* [95]. However, large clinical trials with prasugrel and ticagrelor did not show a clinical benefit and found no reduction in painful events and VOE in pediatric and adult patients with SCD [[96], [97], [98], [99]]. The clinical trials targeting platelets relied on patient-reported painful crises and hospitalization rates but did not capture markers of vascular injury, coagulation, inflammation, and organ damage.

Recent research into nontraditional pathways of platelet activation in SCD may reveal why these traditional antiplatelet agents were not effective clinically. One possible effector is HMGB1, which is elevated in SCD and activates platelets via TLR4 signaling. This pathway enhances ADP release and increases platelet surface P2Y_12_ expression, effectively priming platelets for purinergic signaling [85]. Paradoxically, SCD platelets demonstrate impaired functional responses to ADP [100]. This apparent contradiction of hyperactivity despite impaired P2Y_12_ signaling suggests the presence of compensatory, noncanonical activation routes. Alternative mechanisms, including signaling through TLR4, PAR1/PAR4, or CLEC2 pathways, may bypass or outweigh conventional ADP-driven aggregation. For example, HMGB1–TLR4 signaling has been shown to activate the NLRP3 inflammasome, resulting in caspase 1 activation and platelet aggregation [101]. Heme also acts through platelet TLR4 to induce mitochondrial reactive oxygen species formation and degranulation [86]. There is also evidence that heme can activate platelet aggregation through CLEC2 [89]. Together, these findings highlight a complex network of platelet activation in SCD that is likely resistant to traditional antiplatelet strategies targeting purinergic signaling alone.

## 4 Thromboinflammation and SCD Complications

Despite its central role in disease progression, the mechanistic foundations of thromboinflammation in SCD remain incompletely understood, and targeted therapies have yet to be successfully translated. Investigating these mechanisms is essential not only for preventing acute VOEs but also for preserving long-term organ function and improving survival. Understanding the context-specific signaling pathways, cellular interactions, and vascular bed vulnerabilities in SCD will be critical for developing rational, mechanism-based interventions that address both the acute and chronic complications of this uniquely aggressive thromboinflammatory disease. In this section, we will introduce how thromboinflammation contributes to the risk of both arterial and venous thromboses, chronic kidney disease, cardiopulmonary complications, and pain.

### 4.1 Venous thrombosis

SCD is recognized as a significant thrombophilia with a high risk of venous thromboembolism (VTE), including both deep vein thrombosis and pulmonary embolism. Epidemiological studies have revealed an incidence of VTE of 11.3% to 25% by the age of 40 years [102,103], along with increased risk of recurrence [104]. Notably, there is a higher incidence of pulmonary embolism than deep vein thrombosis, which may be due to increased embolization or from *in situ* thrombus formation in the lung [103,104]. A history of VTE is a risk factor for mortality, as well as other complications including chronic kidney disease and PH [103,104]. Importantly, the use of central venous access devices, common in patients with frequent hospitalizations or chronic transfusions, may further increase the risk of thrombosis [102]. Work in our laboratory has implicated both TF and FXII-dependent thrombin generation in models of venous thrombosis in sickle mice [35].

### 4.2 Arterial thrombosis and stroke

Stroke is one of the most devastating complications of SCD. By age of 40 years, 10% to 20% of patients have a cerebrovascular event. Both overt strokes and silent cerebral infarcts, which occur more frequently, are driven by the chronic thromboinflammatory environment [105]. Preclinical models have identified several critical mediators of stroke risk in SCD. We recently demonstrated that FXII contributes to stroke incidence and severity in a mouse model of SCD [35]. Moreover, neutrophils contribute to stroke outcomes by releasing NETs and other proinflammatory mediators that recruit platelets [[106], [107], [108]]. Dysregulation of the EPCR-PAR1 pathway directly contributes to microvascular thrombus formation in the cerebral microvasculature [39]. This is particularly interesting given the potential for 3K3-APC to treat stroke in the population without SCD [109]. Together, these preclinical findings highlight the complex interplay among coagulation, inflammation, and vascular dysfunction in the pathogenesis of stroke in SCD.

### 4.3 Chronic kidney disease

The microvascular occlusions caused by sRBCs, platelets, and neutrophils disrupt renal blood flow and lead to glomerular damage. Chronic inflammation and oxidative stress, mediated by heme and HbS, contribute to endothelial injury and thrombosis within the renal microvasculature. Over time, this results in glomerulosclerosis and chronic kidney disease [110]. The inflammatory environment in the kidneys can further exacerbate glomerular injury, driving a vicious cycle of progressive kidney dysfunction in SCD patients.

Thrombin generation and inflammation have been implicated in renal injury [28] in SCD mice. We have also shown that FXII and its cofactor HK play critical roles in the development of sickle nephropathy [34,35]. HK, a precursor to bradykinin, is aberrantly activated in SCD, promoting both hypercoagulability and inflammation [34]. In SCD mouse models, we have shown that HK or FXII deficiency improve renal function, indicated by reduced albuminuria, glomerulosclerosis, inflammatory cell infiltration, iron deposition, and interstitial fibrosis, with preservation of brush border thickness and podocytes [34,35]. These findings underscore HK and FXII as key mediators of thromboinflammation and kidney injury in SCD, highlighting potential therapeutic targets.

Sickle nephropathy may also be mediated by thrombin-PAR1 signaling. A recent study demonstrated that chronic hemolysis leads to loss of EPCR from the kidney vasculature, contributing to endothelial dysfunction and renal injury [40]. Maintaining EPCR expression in sickle mice ameliorates kidney injury, emphasizing the critical role of the EPCR-PAR1 axis in maintaining vascular homeostasis [40].

Angiogenic dysregulation is increasingly recognized as a driver of renal and other end-organ complications in SCD. Hypoxia, driven by recurrent VOE and impaired microvascular blood flow, acts as a strong inducer of the transcription factor hypoxia inducible factor 1α, which upregulates VEGF and PlGF, key mediators of vascular remodeling. While the contribution of angiogenic factors to sickle cell nephropathy remains underexplored, emerging studies suggest their significant involvement in renal pathology. Acute intravascular hemolysis rapidly increases VEGF production, promoting endothelial cell migration and neovascularization [111]. Genetic polymorphisms in VEGF are linked to increased vascular permeability within the renal microvasculature, promoting aberrant angiogenesis and glomerular injury [112]. Elevated PlGF also stimulate ET-1 production, worsening vascular dysfunction, inflammation, and progressive renal damage in SCD [113]. The complex interplay among heme-induced hypoxia, angiogenesis, and vascular injury in SCD may offer novel therapeutic targets aimed at preserving kidney function and improving overall outcomes in affected patients.

### 4.4 Cardiopulmonary complications

Thromboinflammation is a central driver of cardiopulmonary complications in SCD, particularly PH and ACS, both major contributors to morbidity and mortality. Intravascular hemolysis releases cell-free hemoglobin and heme, which scavenge NO a critical vasodilator, resulting in vasoconstriction and impaired vascular tone [114]. Chronic hemolysis also promotes systemic and pulmonary inflammation through the release of proinflammatory cytokines.

Endothelial activation, characterized by upregulation of VCAM-1 and intracellular adhesion molecule 1, facilitates the adhesion of leukocytes and platelets, aggravating vascular injury. This proinflammatory, prothrombotic environment enhances TF expression, thrombin generation, and platelet activation. Indeed, adhesion of sRBCs and platelets to the endothelium and promote thrombus formation, contributing directly to the pathogenesis of PH [84]. Platelets trigger NET release, further obstructing the pulmonary microvasculature and drive acute lung injury [[115], [116], [117], [118]].

ACS, one of the most life-threatening complications of SCD, presents with acute chest pain, fever, hypoxemia, and pulmonary infiltrates. It results from pulmonary vaso-occlusion, triggered by sickled cells, infection, or fat embolism within the pulmonary vasculature, leading to alveolar damage and microthrombus formation, ventilation-perfusion mismatch, and hypoxemia [119]. Approximately 50% of patients with SCD will experience at least 1 episode of ACS in their lifetime [120]. Although human studies on inflammatory mechanisms in ACS remain limited [121], available evidence suggests that sRBCs, the activated endothelium, and inflammatory mediators synergize to drive pulmonary injury.

ACS may also lead to PH [119,122] as repeated episodes of ACS cause chronic lung injury, fibrosis, hypoxic vasoconstriction, and vascular remodeling. This chronic pulmonary insult promotes pulmonary vasculopathy, endothelial damage, and *in situ* thrombosis—key contributors to PH development [123,124]. Diagnosis of PH in SCD remains challenging due to its multifactorial etiology and overlapping symptoms with ACS and other pulmonary complications. The underlying pathophysiology includes endothelial injury, chronic inflammation, hypercoagulability, chronic hemolysis, and altered levels of NO and ET-1, which have the opposing effects [119].

Accumulating evidence highlights the role of dysregulated angiogenic signaling in SCD-associated PH. Studies in sickle mouse models have demonstrated that free heme promotes expression of PlGF and IL-6, leading to increased ET-1 production and vascular remodeling [125,126]. These pathways not only drive PH but are also implicated in renal and cardiac dysfunction [127]. Elevated angiogenic and inflammatory mediators correlate with higher right ventricular systolic pressure and right ventricular hypertrophy in mouse models, underscoring the systemic impact of thromboinflammation [125].

### 4.5 Pain

Pain is the most prevalent and debilitating symptom of SCD and a leading cause of hospitalization. Although opioids remain the standard treatment, their risks underscore the need for safer long-term pain management. Acute pain is linked to vaso-occlusive events, whereas chronic pain is attributed to recurrent VOE, release of extracellular traps and enzymes from neutrophils (elastase) and mast cells (tryptase), and subsequent sensitization of peripheral nerve fibers that promotes neuroinflammation. Mechanisms of SCD-related pain have been well described by others [128,129].

A direct link between coagulation and acute pain has not been established, yet activation of PAR2 on peripheral nerve fibers via elastase and tryptase sensitizes neurons to pain signals. Crizanlizumab, an anti–P-selectin antibody, reduces the frequency of painful events [130] and biomarkers of P-selectin–mediated adhesion correlate with patient-reported pain in the Evaluation of Longitudinal Pain Study in Sickle Cell Disease study [131]. That same study also demonstrated elevated levels of cytokines (IL-6 and C-reactive protein) and coagulation biomarkers (Thrombin-AntiThrombin complexes and D-dimer) during self-reported painful episodes. Extracellular vesicles derived from endothelial cells are also elevated during self-reported painful events [132], consistent with prior findings that TF-positive endothelial extracellular vesicles increase during crisis compared with steady state in sickle patients [133]. Recently, the complement system has been implicated in VOEs, as well as in acute (eg, triggered by cold and hypoxia) and chronic pain in preclinical SCD models [45,[134], [135], [136]].

## 5 Summary of SCD as A Thromboinflammatory State

SCD presents a distinct form of thromboinflammation, characterized by chronic hemolysis, sustained endothelial activation and multicellular aggregate formation, all driving recurrent VOE and progressive end-organ damage. Unlike other thromboinflammatory states that are episodic, such as COVID-19, SCD involves persistent inflammation fueled by ongoing intravascular hemolysis. Free heme and HbS act as DAMPs to activate TLR4 and other receptors on endothelial cells, monocytes, and neutrophils to promote cytokine release, adhesion molecule expression, and TF upregulation. sRBCs, hyperreactive platelets, and activated neutrophils converge on this activated endothelium to form multicellular aggregates that obstruct the vasculature. Each cell type not only contributes to inflammatory signaling but also amplifies thrombin generation, resulting in a vicious cycle of coagulation and inflammation. This uniquely multicellular, sustained thromboinflammatory state targets vulnerable low-flow microvascular beds in the bone marrow, lung, kidney, spleen, and brain, leading to cumulative end-organ damage. Understanding the cellular and molecular pathways fueling thromboinflammation is essential for breaking this pathological cycle and mitigating the lifelong burden of this disease.
